# Distinct Lysosome Phenotypes Influence Inflammatory Function in Peritoneal and Bone Marrow-Derived Macrophages

**DOI:** 10.1155/2014/154936

**Published:** 2014-12-23

**Authors:** Kassandra Weber, Joel D. Schilling

**Affiliations:** ^1^Diabetic Cardiovascular Disease Center, Washington University School of Medicine, St. Louis, MO 63110, USA; ^2^Department of Medicine, Washington University School of Medicine, St. Louis, MO 63110, USA; ^3^Department of Pathology and Immunology, Washington University School of Medicine, St. Louis, MO 63110, USA

## Abstract

Lysosomes play a critical role in the degradation of both extracellular and intracellular material. These dynamic organelles also contribute to nutrient sensing and cell signaling pathways. Macrophages represent a heterogeneous group of phagocytic cells that contribute to tissue homeostasis and inflammation. Recently, there has been a renewed interest in understanding the role of macrophage autophagy and lysosome function in health and disease. Thioglycollate-elicited peritoneal and bone marrow-derived macrophages are commonly used ex vivo systems to study primary macrophage function. In this study, we reveal dramatic baseline differences in the lysosome morphology and function between these macrophage populations and provide evidence that these differences can be functionally relevant. Our results provide important insights into the diversity of lysosomes in primary macrophages and illustrate the importance of accounting for this in data interpretation.

## 1. Introduction

The lysosome is a dynamic organelle that operates at an acidic pH and contains numerous enzymes that are critical for cellular degradation pathways. Uptake of extracellular material reaches the lysosome via the endocytic pathway, whereas intracellular cargo is delivered to the lysosome via autophagy [1]. The lysosome can also play a role in secretion, membrane repair, and cell clearance through the process of lysosomal exocytosis [2, 3]. Recently, the importance of lysosomes in cell signaling pathways and nutrient sensing has also become apparent [4, 5]. Importantly, the regulation of lysosome structure and function is cell type dependent and is regulated by environmental stimuli.

Macrophages are cells of the innate immune system that are important for organ homeostasis, inflammation, host defense, and tissue repair [6]. Recently, there has been a renewed interest in macrophage lysosome biology. The importance of macrophage autophagy in several clinically relevant diseases has helped to fuel this renaissance [7–10]. Moreover, it has also come to light that lysosomal pathways activate IL-1*β* release via the inflammasome in several important human diseases including atherosclerosis, gout, and Alzheimer's disease [11–13]. Also, of relevance, lipid overload and obesity can also induce “lysosomal reprogramming” in adipose tissue macrophages, which may contribute to the metabolic complications of nutrient excess [14]. Together, these and many other studies indicate that additional cellular and molecular studies of lysosome function in macrophages will be critical to understand the role of this organelle in inflammatory diseases.

Ex vivo analysis of primary macrophages will be important for mechanistic cell biology experiments investigating lysosome function in phagocytic cells. The most common sources of primary macrophages include bone marrow-derived macrophages (BMDMs) and thioglycollate- (TG-) elicited peritoneal macrophages (pMACs). Although BMDMs and pMACs are derived from very distinct environments they are often used interchangeably to model macrophage biology. pMACs are monocyte derived cells that are typically isolated from the peritoneal cavity 3–5 days after TG administration [15]. Thus, pMACs are actively involved in the process of inflammation resolution, which includes the uptake of dead cells and/or debris through efferocytosis or phagocytosis, respectively. In contrast, BMDMs are derived from a myeloid precursor cell functionally naïve and would be expected to have fewer demands on their endosomal system. Based on this, we hypothesized that the lysosomes would be phenotypically and functionally distinct in these subtypes of primary macrophages.

In the current study, we investigated lysosome content, morphology, and function in pMACs and BMDMs. Our data demonstrate that pMACs have a larger lysosome volume, increased cathepsin activity, and enhanced expression of several lysosomal genes and proteins. Moreover, using the example of the lipotoxic inflammasome, we provide evidence that these differences in the lysosomal compartment can influence macrophage inflammatory responses. Together, our results argue that the interpretation of data involving lysosome-dependent processes in primary macrophages must take the source of the cells into consideration.

## 2. Materials and Methods

### 2.1. Reagents

CAO74-ME and bafilomycin A were from Enzo Life Sciences. Lysotracker red and TMR-dextran (10,000 MW) were from Invitrogen. The cathepsin B activity assay was from Immunocytochemistry Technologies. Ultrapure* E. coli* LPS was from Invivogen. Thioglycollate was from Difco. Fatty acids were from Nu-Chek Prep. The cathepsin D and LAMP1 antibodies were from Abcam. The actin antibody was from Sigma-Aldrich. The CD107a (LAMP-1) PE conjugated antibody was from eBiosciences (cat#12-1071). The ATG5 antibody was from Novus Biologics. Ultrapure-bovine serum albumin (BSA) was from Lampire and was tested for TLR ligand contamination prior to use.

### 2.2. Cell Culture

Peritoneal macrophages (pMACs) were isolated from C57BL/6 mice 4 days after intraperitoneal injection of 3.85% thioglycollate and plated at a density of 0.9-1 × 10^6^ cells/mL in DMEM containing 10% inactivated fetal serum (IFS), 50 U/mL penicillin G sodium, and 50 U/mL streptomycin sulfate (pen-strep), 2 mM L-glutamine, and sodium pyruvate. Stimulations were performed on the day after harvest. Bone marrow-derived macrophages (BMDMs) were prepared by harvesting bone marrow from the femurs and tibias of 8–12-week-old C57BL/6 mice. The cells were seeded in 10 cm dishes and differentiated for 6 days in DMEM media as above supplemented with 10% supernatant from CMG14.12 cells as a source of M-CSF [16]. On day 6, BMDMs were plated at 1 × 10^6^ cells/mL in media containing 5% supernatant from CMG14.12 cells. Stimulations were performed on the day after harvest in media containing 5% CMG1.12 media. For flow cytometry experiments, macrophages were cultured on low adherence plates (Greiner Bio-One) to facilitate cell harvest. Cells were removed from the plate by washing with PBS followed by 10 minutes with Cell Stripper (GIBCO) and then 10 minutes with EDTA/trypsin (Sigma). Growth medium was supplemented with palmitate and BSA at a 2 : 1 molar ratio as described previously [17] and BSA-supplemented media were used as control. For cell stimulations, PBS or LPS (50 ng/mL) were added to BSA or free fatty acid containing media. Flow cytometry was performed on a BD Biosciences FACSCalibur machine and the data was analyzed using Flowjo software.

### 2.3. Mice

Wild type (WT) C57BL/6 mice were obtained from Oriental Bioscience and maintained in our mouse colony. The ATG5flox X LysM-Cre on the C57BL/6 background were kindly provided by Skip Virgin (Washington University). Mice were maintained in a pathogen-free facility on a standard chow diet ad libitum (6% fat). All animal experiments were conducted in strict accordance with NIH guidelines for humane treatment of animals and were reviewed by the Animal Studies Committee of Washington University School of Medicine.

### 2.4. RNA Isolation and Quantitative RT-PCR

Total cellular RNA was isolated using Qiagen RNeasy columns and reversely transcribed using a high capacity cDNA reverse transcription kit (Applied Biosystems). Real-time qRT-PCR was performed using SYBR green reagent (Applied Biosystems) on an ABI 7500 fast thermocycler. Relative gene expression was determined using the delta-delta CT method normalized to 36B4 expression. Mouse primers sequences were as follows (all 5′-3′):* 36B4* (forward-ATC CCT GAC GCA CCG CCG TGA, reverse-TGC ATC TGC TTG GAG CCC ACG TT);* ctsB* (forward-GAT CAA GGA CCA CCA CAT CC, reverse-CTT AGG AGT GCA CGG GAG AG);* ctsD *(forward-GAC AGC TCC CCG TGG TAG TA, reverse-CAA CAG AAG CTG GTG GAC AA);* ctsK *(forward-TGC CGT GGC GTT ATA CAT AC, reverse-CGG CTA TAT GAC CAC TGC CT);* LAMP1* (forward-TCT TCA GTG TGC AGG TCC AG, reverse-ATG AGG ACG ATG AGG ACCAG);* LAMP2A* (forward-CCA AAT TGG GAT CCT AAC CTA, reverse-TGG TCA AGC AGT GTT TAT TAA TTC C);* LAMP2B* (forward-GGT GCT GGT CTT TCA GGC TTG ATT, reverse-ACC ACC CAA TCT AAG AGC AGG ACT):* ATP6V1H* (forward-AGA CAG CCA GCA ACA CAC TG, reverse-TCA CAG AAA CTT CGT GGC AG);* p62* (forward-GCT GCC CTA TAC CCA CAT CT, reverse-CGC CTT CAT CCG AGA AAC);* LC3* (forward-CGT CCT GGA CAA GAC CAA GT, reverse-ATT GCT GTC CCG AAT GTCTC).

### 2.5. Western Blotting

Total cellular protein was isolated by lysing cells in 150 mM NaCl, 10 mM Tris (pH 8), triton X-100 1%, and 1X Protease Complete. Proteins were separated on a TGX gradient gel (4–20%; Biorad) and transferred to a nitrocellulose membrane. Western blotting for cathepsin D and actin was performed using 40 *μ*g of total cellular protein.

### 2.6. LAMP1 Flow Cytometry

After the indicated stimulations, pMACs were removed from the plate as described above and fixed in 4% PFA for 15 minutes in the dark at room temperature. After fixation, cells were permeabilized (PBS + 0.1% Triton X) for 15 minutes in the dark and washed with FACs buffer + 0.1% triton X. Cell were treated with Fc receptor block (BD Pharmigen) for 5 minutes on ice followed by incubation with CD107a (LAMP1) PE antibody for 30 minutes in the dark on ice. Cells were washed in FACs buffer followed by flow cytometric analysis. 

### 2.7. Lysosome Imaging

After the indicated stimulations, cells were removed from the plate as described above and then stained with 500 nM lysotracker red in tissue culture media for 15 minutes at 37°C. After staining, cells were washed three times with PBS, harvested as described above, and analyzed by flow cytometry. For fluorescent dextran experiments, macrophages were incubated with 500 *μ*g/mL tetramethylrhodamine- (TMR-) dextran for 2 h in regular media. For immunofluorescence microscopy, macrophages were plated on sterile glass coverslips followed by staining. Lysotracker red or TMR-dextran stained cells were fixed for 10 minutes in 4% paraformaldehyde followed by nuclear staining with Hoechst 33342 dye. The cover slips were mounted on glass slides and imaged using a Zeiss confocal microscope.

### 2.8. Cathepsin B Activity Flow Cytometry

After the indicated stimulations, pMACs were removed from low adherence plates as described above. The fluorescently labeled cathepsin B substrate was reconstituted per manufacturer's instructions and diluted in DMEM+10% IFS. Macrophages were incubated in 150 *μ*L of staining solution for 45 minutes at 37°C under 5% CO_2_ with gentle mixing every 10 minutes. After the incubation, cells were washed 2 times in 1 mL of PBS and analyzed by flow cytometry (FL2).

### 2.9. IL-1*β* ELISA

Supernatants were harvested from macrophage cultures after the indicated stimulations. IL-1*β* was quantified using a DuoSet ELISA kit (R&D Systems) according to the manufacturer's instructions.

### 2.10. Statistics

Statistical analysis was performed using GraphPad Prism software. All results are expressed as means ±SE. Groups were compared by paired Student's *t*-test. A value of *P* ≤ 0.05 was considered significant. 

## 3. Results/Discussion

### 3.1. Lysosome Content and Activity Are Enhanced in pMACs Compared to BMDMs

**Figure 1:**
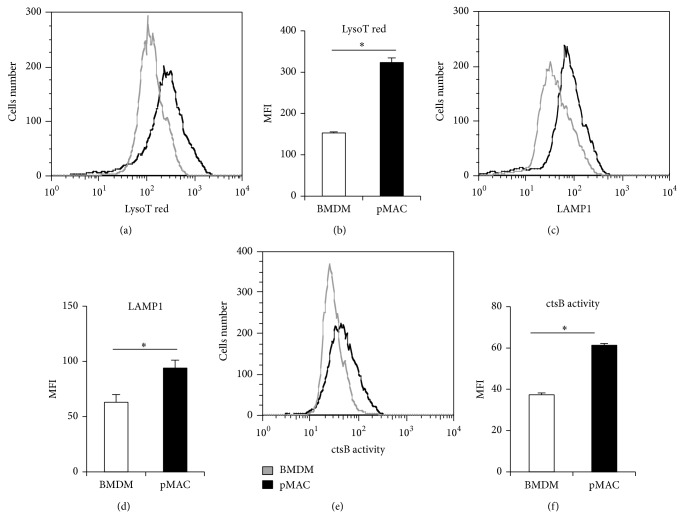
Peritoneal macrophages have increased lysosome volume and activity compared to BMDMs. (a, b) Thioglycollate-elicited peritoneal macrophages (pMACs) or bone marrow-derived macrophages (BMDMs) were stained with lysotracker red and analyzed by flow cytometry. A representative histogram (a) and FL2 mean fluorescence intensity (MFI) quantification (b) are shown. (c, d) Total LAMP1 was determined by staining fixed and permeabilized pMACs or BMDMs with a LAMP1 (CD107a) PE antibody followed by flow cytometry assessment. A representative histogram (c) and MFI quantification (d) are shown. (e, f) Cathepsin B activity was assessed using a fluorogenic cathepsin B substrate that requires cleavage to produce fluorescent signal. After incubation with substrate, pMACs and BMDMs were analyzed by flow cytometry. A representative histogram (e) and MFI quantification (f) are shown. Bar graphs report the mean ± standard error (SE) for a minimum of 3 experiments, each performed in triplicate. ^*^ *P* < 0.05 for pMAC versus BMDM.

To investigate differences in the lysosome compartment of pMACs compared to BMDMs, we quantified lysosome volume in these two sets of primary macrophages using the lysosomotropic dye lysotracker red coupled with flow cytometric analysis. Using this approach, lysosome content was significantly increased in pMACs relative to BMDMs (Figures 1(a) and 1(b)). In line with this finding, pMACs also had significantly higher levels of the lysosomal membrane protein LAMP1 compared to BMDM (Figures 1(c) and 1(d)). Together, these findings support the presence of an expanded lysosomal compartment in pMACs. To assess lysosomal protease function, we analyzed the activity of cathepsin B using the fluorogenic substrate magic red. As shown in Figures 1(e) and 1(f), cathepsin B activity was increased in pMACs when compared to BMDMs.

### 3.2. Distinct Lysosome Size and Morphology in pMACs Compared to BMDMs

**Figure 2:**
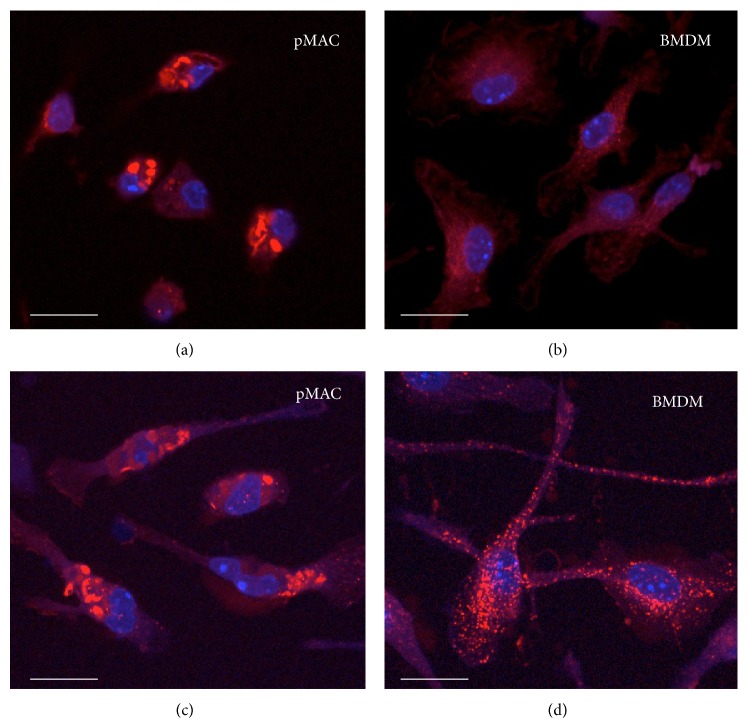
pMACs and BMDMs have distinct lysosome morphology. (a–d) pMACs (a, c) or BMDMs (b, d) were incubated with lysotracker red (a, b) or TMR-dextran (c-d) to label lysosomes. Nuclei are stained with Hoechst (blue). Representative immunofluorescent images are shown. The white bar indicates 10 microns.

Lysosomes have several distinct morphologic appearances that can vary based on cell type and activation status including spheroid, ovoid, or tubular. In addition, the size of lysosomes can vary dramatically [18]. To visualize lysosomes in pMACs and BMDMs, we utilized two complementary immunofluorescent approaches: (1) lysotracker red staining and (2) tetramethylrhodamine- (TMR-) dextran staining. Lysotracker red freely crosses cell membranes and is concentrated in acidic organelles whereas TMR-dextran is taken up via endocytosis and reaches lysosomes in ~30 min. Lysosome size and morphology were similar using both staining techniques (Figure 2). Strikingly, pMACs had much larger lysosomes than BMDMs. In addition, the morphology was more frequently ovoid and tubular in pMACs and spherical in BMDMs (Figures 2(a)–2(d)). Notably, pMAC lysosome morphology is similar to that described from adipose tissue macrophages during high fat feeding [14]. Stimulation of BMDMs or pMACs with LPS did not lead to a significant change in lysosome morphology (data not shown).

### 3.3. Lysosomal Gene and Protein Expression Are Distinct in pMACs Compared to BMDMs

**Figure 3:**
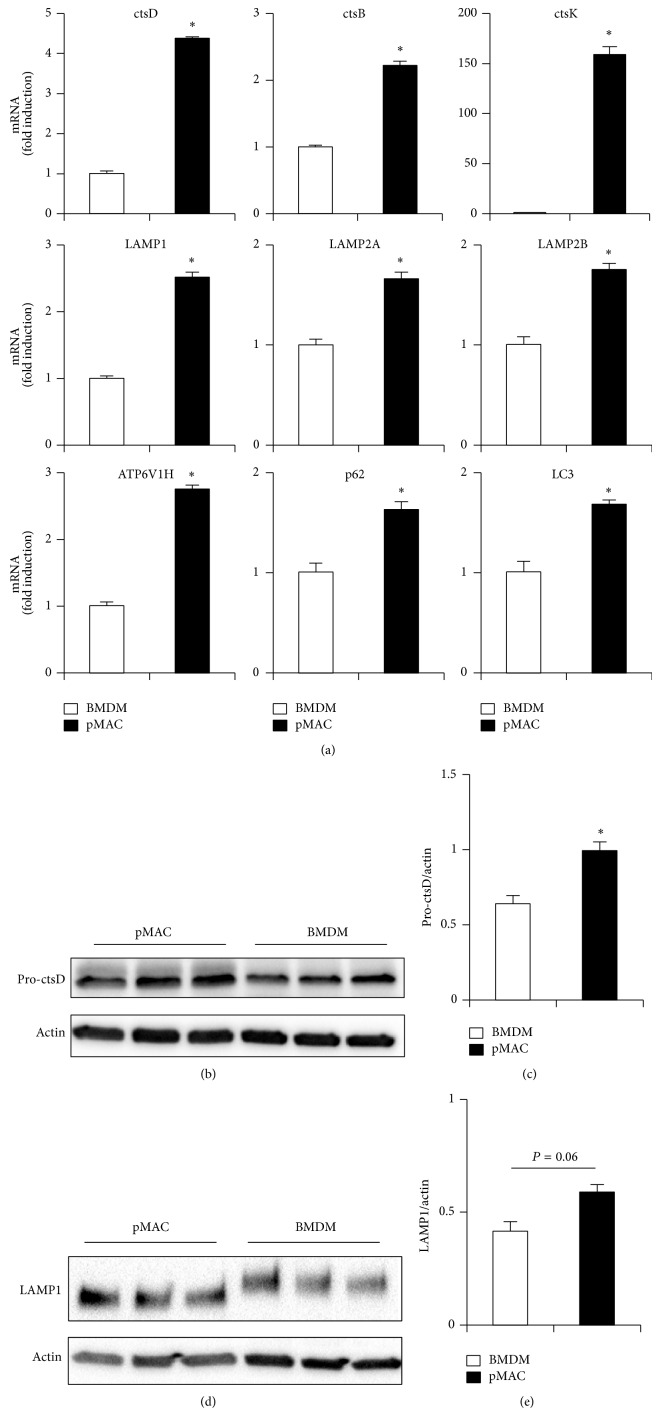
Expression of lysosomal genes is enhanced in pMACs. (a) RNA was isolated from pMACS and BMDMs and expression of the indicated lysosome and autophagy genes was determined by qRT-PCR. Expression was normalized to 36B4. (b, d) Total cellular protein was isolated from pMACs or BMDMs and expression of pro-cathepsin D (ctsD; (b)) or LAMP1 (d) was determined by western blotting. (c, e) Quantification of pro-cstD protein (45 kD) expression (c) or LAMP1 (110–120 kD) (e) normalized to actin. Bar graphs report the mean ± SE for a minimum of 2 experiments, each performed in triplicate. ^*^ *P* < 0.05 for pMAC versus BMDM.

Emerging data indicates that lysosome biogenesis and function are regulated in part at the transcriptional level [19]. Therefore, we compared the expression of several lysosome and autophagy related genes in BMDMs and pMACs. Consistent with the flow cytometry and imaging data, the expression of genes encoding cathepsin proteases, lysosomal membrane proteins, and the vacuolar ATPase was increased in pMACs relative to BMDMs (Figure 3(a)). In addition, the autophagy related genes LC3 and p62 were also expressed at a higher level in pMACs (Figure 3(a)). Thus, mRNA expression of lysosome and autophagy related genes is increased in pMACs, which is likely related to the expanded lysosome compartment observed in these primary macrophages. At the protein level, pro-cathepsin D protein content was increased in pMACs relative to BMDMs (Figures 3(b) and 3(c)). Similar to the flow cytometry data, LAMP1 protein content trended towards an increase in pMACs compared to BMDMs (Figures 3(d) and 3(e)). Interestingly, the molecular weight of LAMP1 was significantly greater in BMDMs suggesting a higher level of glycosylation, a finding which could further affect lysosome function and stability in these two types of primary macrophages (Figure 3(d)).

### 3.4. The Differences in Lysosome Phenotype Translate into Distinct Mechanisms for Activation of the Lipotoxic Inflammasome

**Figure 4:**
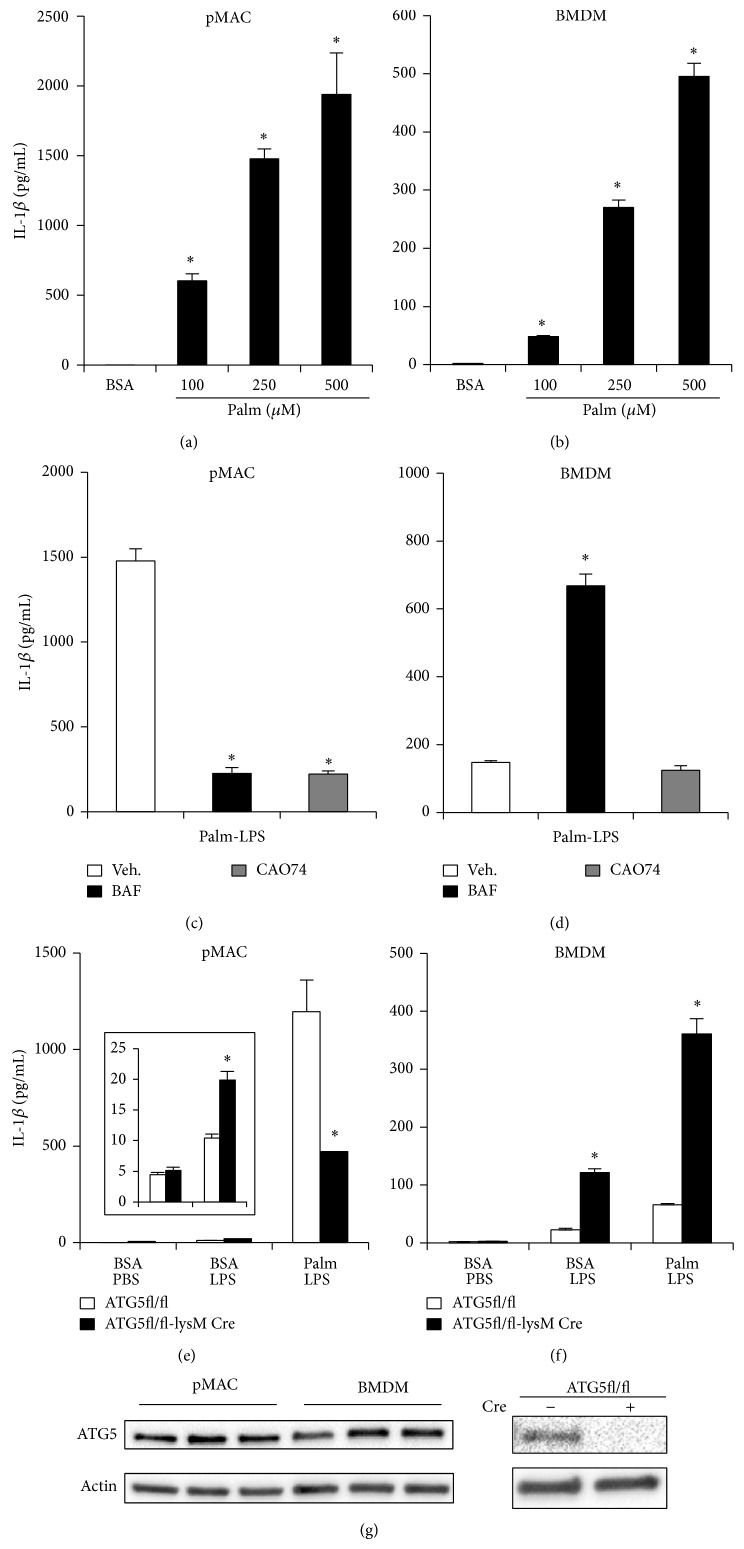
Differential role of the lysosome during activation of the lipotoxic inflammasome in pMACs compared to BMDMs. (a, b) pMACs (a) or BMDMs (b) were preloaded with the indicated doses of palmitate (palm) or with BSA for 2 h followed by BSA or palm ±50 ng/mL LPS for 20 h after which IL-1*β* in the supernatant was quantified by ELISA. (c, d) pMACs (c) or BMDMs (d) were stimulated with palmitate and LPS in the presence of bafilomycin (BAF, 25 nM) or CAO74-ME (10 *μ*M) and IL-1*β* release at 20 h was determined by ELISA. (e, f) pMACs (e) or BMDMs (f) were prepared from WT (white bars) or ATG5KO (black bars; ATG5fl/fl X LysM-Cre) mice and were subsequently stimulated with BSA-PBS, BSA-LPS, or palm-LPS for 20 h. IL-1*β* release was quantified by ELISA. The inset in (e) shows data for BSA-PBS versus BSA-LPS with an adjusted scale. (g) Total cellular protein was isolated from pMACs or BMDMs and expression of ATG5 was determined by western blotting (*left panel*). As a control for the antibody, protein was also isolated from WT and ATG5KO pMACs and analyzed by western blot (*right panel*). Bar graphs report the mean ± standard error (SE) for a minimum of 3 experiments, each performed in triplicate. ^*^ *P* < 0.05 for BSA-PBS versus palm-LPS; vehicle versus inhibitor; or WT versus KO.

To investigate whether the observed differences in lysosome phenotype modulate macrophage inflammatory responses, we explored the lipotoxic inflammasome as a model system. We, and others, have shown that the combination of LPS and the saturated fatty acid (SFA) palmitate triggers the release of IL-1*β* through an NLRP3 and caspase 1-dependent mechanism [20–22]. In prior studies using this system, it has been shown that IL-1*β* release from pMACs is dependent on lysosome damage and cathepsin proteases, whereas the role of lysosomes in BMDMs is less clear [21]. To directly compare pMACs and BMDMs using this system, we simultaneously stimulated both cell types with LPS in the presence of increasing concentrations of palmitate and the release of IL-1*β* was quantified. Both pMACs and BMDMs secreted IL-1*β* in a dose dependent fashion; however, pMACs consistently secreted 5–10 times more cytokines than BMDMs despite using a similar number of cells (Figures 4(a) and 4(b)). Importantly, FA uptake was similar between the pMACs and BMDMs (data not shown). Consistent with prior studies, IL-1*β* release from pMACs was almost completely prevented by the cathepsin B inhibitor, CAO74, or lysosomal acidification inhibitor, bafilomycin (BAF), in palmitate-LPS treated pMACs (Figure 4(c)). This was not the case in BMDMs where IL-1*β* release was unaffected by CAO74 and actually increased with BAF (Figure 4(d)). Thus, lysosome inhibitors differentially influence lipotoxic inflammasome activation in pMACs compared to BMDMs, an observation that may account for some of the discrepancies between prior publications [20–22]. More importantly, these findings argue that the baseline differences in lysosome content and function between pMACs and BMDMs can alter the response of these cells to inflammatory stimuli.

Lysosomes are also important for the degradation of autophagic cargo, which can also modulate the inflammasome response [23]. Therefore, using ATG5 KO cells, we tested the impact of autophagy deficiency on activation of the lipotoxic inflammasome in pMACs versus BMDMs. As can be seen in Figures 4(e) and 4(f), the phenotypes observed using autophagy deficient cells were different in these primary macrophage systems. In pMACs, ATG5 KO cells had a modest increase in IL-1*β* release after LPS treatment (Figure 4(e) *inset*), but IL-1*β* secretion following palmitate-LPS stimulation was reduced compared to WT cells (Figure 4(e)). In distinction, ATG5 KO BMDMs released more IL-1*β* in response to both LPS and palmitate-LPS treatment (Figure 4(f)). This was not related to differences in ATG5 protein expression, which was similar between the two types of macrophages (Figure 4(g)). Instead, the development of lysosome dysfunction in pMACs treated with palmitate-LPS may explain why genetic autophagy deficiency does not further enhance inflammasome activation [24]. In contrast, ATG5 KO BMDMs phenocopy cells treated with BAF, which also inhibits autophagic flux. These findings argue that genetic or pharmacologic inhibition of autophagy in BMDMs unmasks an important suppressive role for this degradative process on lipotoxic inflammasome activation. Further investigation will be necessary to evaluate the mechanisms that account for these differential phenotypes. Interestingly, recent evidence suggests that autophagy can increase lysosome activity, which could be particularly important for BMDMs as they have less active lysosomes at baseline [25, 26]. Taken together, this data suggests that pMACs and BMDMs respond to lipotoxic stimulation through fundamentally different mechanisms, and at least part of these differences is related to distinct lysosome phenotypes.

The findings presented in this paper illustrate several significant differences between lysosomes in pMACs and BMDMs. Specifically, pMACs display enhanced lysosome volume, size, and cathepsin activity. The “activated” lysosome compartment in pMACs may reflect the signals they receive in the inflammatory setting of peritonitis. In contrast, BMDMs are naïve macrophages that have not encountered “activation” signals. Using the example of the lipotoxic inflammasome, we also provide evidence that the distinct lysosome phenotypes observed in BMDMs and pMACs can influence macrophage inflammatory function. Thus, when conducting experiments of lysosome function with primary macrophages, it is critical to account for baseline lysosome phenotype to ensure appropriate interpretation of data. We would argue that the choice of BMDM versus pMAC for experimentation should be tailored to the specific research question, and a low threshold should exist for comparing multiple macrophage populations. In addition, further comparative analysis of lysosomes between pMACs, BMDMs, and other in vivo isolated macrophages will be necessary to shed additional light on the dynamic regulation of lysosomes in these important leukocytes.

## Abbreviations

TG: Thioglycollate
BMDM: Bone marrow-derived macrophage
pMAC: peritoneal macrophage.
